# Threats to Belonging—Stressful Life Events and Mental Health Symptoms in Aging Men—A Longitudinal Cohort Study

**DOI:** 10.3389/fpsyt.2020.575979

**Published:** 2020-12-02

**Authors:** Jutta Lindert, Lewina O. Lee, Marc G. Weisskopf, Martin McKee, Susanne Sehner, Avron Spiro

**Affiliations:** ^1^Department of Health and Social Work, University of Applied Sciences Emden, Emden, Germany; ^2^Women‘s Research Center, Brandeis University, Waltham, MA, United States; ^3^Department of Psychiatry, Boston University School of Medicine, Boston, MA, United States; ^4^National Center for Posttraumatic Stress Disorder, Veterans Affairs Boston Healthcare System, Boston, MA, United States; ^5^Department of Environmental Health, Harvard T. H. Chan School of Public Health, Boston, MA, United States; ^6^London School of Hygiene & Tropical Medicine, London, United Kingdom; ^7^Institute for Medical Biometry and Epidemiology, Institute for Epidemiology and Statistics, University of Hamburg, Hamburg, Germany; ^8^Department of Epidemiology, Boston University School of Public Health, Boston, MA, United States

**Keywords:** stressful life events, belonging, aging, mental health, men

## Abstract

**Objectives:** Stressful life events, especially relationship events, are frequent in adult life. We investigated the impact of a variety of stressful life events on symptoms of depression, anxiety, and hostility.

**Methods:** We analyzed data from a large prospective cohort study of men (*n* = 1,437) in the Boston area (assessed in 1985, 1988, and 1991). Main outcomes were measures of depression, anxiety and hostility symptoms. We used the Elders Life Stress Inventory (ELSI) to measure stressful life events in the past 12 months and examine their association with symptoms of depression, anxiety and hostility. First, we analyzed the association of stressful life events with symptom changes; second, we categorized stressful life events into finance/work, health, relationships, loss, living situations events; and third, we estimated the specific association between relationship events and depression, anxiety and hostility symptoms using multilevel models.

**Results:** The most frequent stressful life events were health, relationship, and financial events. Depression, anxiety, and hostility symptoms were relatively stable among men who did not experience these life events. However, those who reported life events in the past 12 months had a greater increase in symptoms of depression (+0.05; 95% CI: 0.01 to 0.10) and of hostility (+0.05; 95% CI: 0.01 to 0.09) than those who did not. Additionally, we found a significant decrease in hostility (−0.05; 95% CI: −0.08 to −0.01) in those experiencing no life events.

**Conclusion:** Relationship events were more important than any other type of events, and were significantly associated with increased depression and hostility in aging men. Although the effects were small, the results point to a need to understand better the impact of relationships on psychopathology in the aging population.

## Introduction

Older people are at particular risk of certain stressful life events (SLEs) such as retirement, loss of loved ones, changes in living situation, and ill health (1, 2). Health, interpersonal, and financial events are especially common (3, 4), and many of these events affect health problems associated with aging (5, 6). Moreover, health- and loss events are especially stressful for older persons, more so than living situation events (2). SLEs in older people are important because they have been linked to depression, anxiety, and hostility (7), three of the leading causes of disease burden in this population. In the Global Burden of Disease (GBD) study the prevalence of major depression in older adults is estimated to be between 3.6 and 4.8%, with many more suffering from clinically relevant depressive symptoms (8). These symptoms are associated with declines in cognition (9, 10) and in physical functioning (11, 12). The rapidly increasing number of older adults worldwide calls urgently for a better understanding of the factors influencing depression-, anxiety- and hostility symptoms in this population (13–15) with estimates that the number of older adults with depression will have doubled by 2050 (16–18).

Despite the diversity of SLEs, few studies have examined the association between different types of events and depression or anxiety. It is known that the number of SLEs is associated with depression, as shown in a meta-analysis of 25 studies (19), although most examined total numbers of SLEs or deaths of significant others only. Other studies point to the relevance of particular SLEs, such as relationship events (20–25). Most research on the association between relationships and health has focused on the positive effects, such as social support, but the effects of negative social interactions have been investigated less. Adverse consequences of relationship-related SLEs have been attributed to biological effects of the resulting stress, such as chronic inflammation and dysregulation in the hypothalamic-pituitary-adrenal (HPA) axis (26, 27).

The present study investigates the impact of different SLEs on depression-, anxiety- and hostility symptoms in older men. Specifically, we aim to (1) describe the number and type of SLEs experienced by aging men, (2) assess the impact of different SLEs on depression, anxiety and hostility symptoms, (3) evaluate the impact of categories of life events on changes in depression, anxiety and hostility, and (4) analyze the effects of relationship events on depression, anxiety and anger symptoms in contrast to the impact of all other events.

Based on a large population-based cohort of men with a mean age of 60.4 (SD = 8.2 years) at the first point when the relevant data were collected (1985), the study asks the following research questions: (1) which stressful life events do aging men experience over time? (2) what impact do individual SLEs have on depression-, anxiety- and hostility symptoms? (3) what impact do different categories of SLE have on depression, anxiety and anger symptoms? and (4) is the risk for depression, anxiety and hostility symptoms higher when relationship events happened in comparison with other events?

## Methods

### Study Design and Participants

The data were taken from the ongoing Veterans Affairs (VA) Normative Aging Study (NAS), which seeks to establish predictors of aging. The NAS was established by the VA in 1963, and 2,280 men from the Greater Boston, Massachusetts (US) area aged 21–80 years were enrolled 1961–1970, based on the absence of any serious mental or physical diseases (28, 29). The current study is based on surviving NAS men surveyed in 1985 (*n* = 1,437), 1988 (*n* = 1,233), and 1991 (*n* = 1,099).

NAS participants were slightly more likely to have a high school diploma than the general US population, and were mostly married at entry into the study (30). For the current study, data are drawn from three mail surveys administered in 1985, 1988, and 1991. Analyses are based on *n* = 1,437 men who completed at least one of the three surveys. Sociodemographic details are given in previous reports on the NAS. Response rates remained high over the years: typically exceeding 80% (28, 29).

### Measurements

#### Outcome Measures

Symptoms of depression, anxiety, and hostility were assessed using subscales of the 90-item Symptom Checklist-90-Revised (SCL-90-R). The SCL-90-R is a widely used scale with good psychometric properties designed to measure self-reported mental health symptoms (31, 32). The depression subscale assesses symptoms of dysphoric mood and affect, signs of withdrawal of life interest, lack of motivation, and loss of vital energy, feelings of hopelessness, and thoughts of suicide. The anxiety subscale assesses nervousness, tension, trembling, and feelings of terror and panic. The hostility subscale reflects cognitive and affective symptoms of aggression, irritability, rage, and resentment. Most men completed the baseline and two follow-up assessments (depression: *n* = 1,014, anxiety: *n* = 1,034, hostility: *n* = 1,024).

#### Independent Variables

Socio-demographic (age, education, professional status) variables were ascertained by self-report. Stressful life events were assessed with the Elders Life Stress Inventory [ELSI (28)]. ELSI is a 30-item self-report scale that assesses occurrence over the past 12 month of events that middle-aged and older adults are likely to experience, such as caretaking for a parent or spouse, or divorce of a child. Each item was answered twice, first responding “yes” or “no” to indicate occurrence, and second rating the impact of the identified items on a 5-point Likert scale (“a little/ somewhat/very much/extremely/ not at all”). The construction of the ELSI utilized a nested strategy—that is, multiple items of increasing severity were organized within a domain (e.g., marital problems, separation from spouse, divorce).

For this study, we considered SLEs both as single events and as event categories. SLEs were categorized into six main categories: financial/work-, health-, relationship-, loss-, living situation-, and marriage (Supplementary Table 1). Finally, we combined SLEs into two main groups: relationship events (i.e., events in the relationships, living situation, and loss categories) vs. non- relationship events (i.e., SLEs in the physical/mental health, and the financial/work categories) in relation to changes in depression, anxiety and hostility. We examined the correlations among the scales using Spearman's correlation coefficient. Variables were moderately correlated (depression and anxiety: 0.53–0.59, depression and hostility: 0.40–0.54, anxiety and hostility: 0.56–0.59).

### Data Analysis

First, we estimated descriptive characteristics of the sample. Descriptive values are presented as means with standard deviation (SD) or absolute frequencies and percentages. Second, to estimate changes in symptoms of depression, anxiety and hostility, we calculated linear regression models. Third, we estimated changes in anxiety, depression and hostility symptoms, accounting for potential missing values and the repeated measurement structure of the data, in a multilevel linear regression model (MLR). In this model, repeated measurements were modeled as random intercept. MLR was adjusted for time of measurement, age at baseline and baseline levels of depression, anxiety and hostility, respectively. We report depression, anxiety and hostility as mean and median symptom levels at each survey year and adjusted differences with corresponding 95% confidence intervals (CI). Fourth, we estimated the effect of age and survey year on the probability of reporting a SLE using multilevel logistic regression models with an iid (independent and identically distributed) -covariance structure (using the xtmelogit command in Stata).

To evaluate effects of SLEs on hostility, anxiety, and depression symptoms, we used the following models: (1) total number of reported SLEs (range = 0–30); (2) any SLE (1 = yes, 0 = no); (3) number of SLE categories with one or more endorsed events (range = 0–6), and (4) each of the 30 SLE items (1 = yes, 0 = no, for each item). Finally, we investigated whether life events predicted changes in depression, anxiety and hostility. To visualize changes in depression, anxiety, and hostility in men with and without events, we plotted figures. Following the standards used in descriptive studies, *p*-values were calculated without correction for multiple testing. Two-sided *p* ≤ 0.05 were considered significant. All analyses were conducted with Stata 14 (Stata Corp. 2015. Stata Statistical Software: Release 14. College Station, TX: StataCorp LP.).

## Results

The number and type of SLEs are displayed Table 1 In 1985, men reported an average of 2.3 SLEs in the past year; in 1988 a mean of 2.9 events; and in 1991 a mean of 3.5 SLEs (Table 2). Participants <60 years reported a mean of 2.9 (SD 2.6) SLEs, those 60–69 years had a mean of 2.8 (SD 2.6), and those 70+ years had a mean of 2.9 (SD 2.4). The mean age at baseline [1985] for this sample was 60.4 years (SD = 8.2 years). At baseline, slightly over half of the men were employed full- or part-time (53 and 2%, respectively); approximately one-third were fully retired (32%), and another 11% were retired but remained involved in part-time work) (Supplementary Table 2). Participants in this study were fairly well-educated; 16% had at least some graduate education, 45% had some college or had graduated college, 37% completed high school, and only 2% did not complete high school. Most (89%) were married, with few reporting being divorced, widowed, or never married. Characteristics of the NAS cohort have been described in detail elsewhere (33).

**Table 1:** Type of stressful life event (SLE) by age group (financial/work -, health -, relationship -, loss -, living situation events and marriage) in the past year.

	**Age categories at baseline**			
	**Total**	**<59**	**60-69**	**≥70**
**Type of SLE^a^**	**% (*n*)**	**% (*n*)**	**% (*n*)**	**% (*n*)**
Any SLE	86.8 (3,328)	86.0 (1,157)	86.8 (1,467)	88.2 (704)
Financial/work events	38.3 (1,462)	41.9 (563)	39.7 (670)	29.0 (229)
Money problems	9.6 (366)	11.0 (147)	8.9 (149)	9.0 (70)
Retirement	14.5 (548)	9.3 (125)	19.4 (324)	12.9 (99)
Decrease in hours worked	9.4 (356)	9.7 (130)	10.0 (166)	7.8 (60)
Increase in hours worked	14.7 (553)	22.5 (301)	11.7 (194)	7.5 (58)
Change to worse job	3.4 (129)	5.5 (73)	2.9 (48)	1.0 (8)
Spouse retired	4.2 (160)	2.7 (36)	5.9 (99)	3.2 (25)
Health events	66.01 (2,530)	58.3 (784)	66.8 (1,128)	77.5 (618)
Deterioration of memory	51.1 (1,942)	40.4 (542)	52.6 (880)	65.9 (520)
Sick or injured	17.1 (649)	14.1 (189)	17.9 (300)	20.5 (160)
Health problem of family member	26.5 (1,006)	25.6 (343)	25.6 (429)	30.0 (234)
Institutionalization of spouse	1.1 (41)	0.5 (7)	1.0 (16)	2.3 (18)
Institutionalization of parent	2.3 (88)	3.7 (49)	2.0 (33)	0.8 (6)
Relationship events	27.3 (1,042)	36.3 (487)	23.8 (400)	19.6 (155)
Divorce	1.1 (42)	1.6 (22)	0.9 (15)	0.6 (5)
Marital separation	1.4 (53)	2.1 (28)	0.9 (15)	1.3 (10)
Troubles with boss or coworkers	10.3 (390)	20.1 (270)	6.7 (112)	1.0 (8)
Worsening relationship with child	8.7 (329)	11.8 (158)	7.6 (128)	5.5 (43)
Worsening relationship with wife	8.9 (335)	10.2 (137)	7.4 (124)	9.5 (74)
Childs divorce	7.3 (275)	6.0 (81)	8.6 (143)	6.6 (51)
Loss events	41.0 (1,568)	38.5 (518)	41.5 (701)	44.2 (349)
Death of spouse	1.7 (64)	0.5 (7)	1.1 (18)	5.0 (39)
Death of son or daughter	0.7 (28)	0.6 (8)	0.8 (13)	0.9 (7)
Death of parent	5.7 (215)	6.7 (90)	5.5 (92)	4.3 (33)
Death of other family member	16.6 (627)	14.0 (187)	18.5 (309)	16.9 (131)
Death of a friend	27.1 (1,022)	22.7 (305)	28.7 (477)	31.4 (240)
Loss of a close friend	3.7 (140)	4.9 (66)	2.6 (43)	4.0 (31)
Living situation related events	32.2 (1,232)	30.7 (413)	30.8 (518)	37.9 (301)
Move to worse residence	1.8 (67)	2.5 (33)	1.6 (26)	1.0 (8)
Deterioration in living conditions	3.8 (146)	4.1 (55)	4.1 (69)	2.8 (22)
Burglarized/robbed	3.6 (138)	4.1 (55)	3.8 (63)	2.6 (20)
Loss of prized possessions	1.1 (40)	1.7 (23)	0.6 (10)	0.9 (7)
Decrease in enjoyed activities	24.1 (917)	18.4 (246)	23.8 (399)	34.6 (272)
Assuming responsibility for parent	6.6 (250)	10.1 (135)	5.7 (96)	2.4 (19)
Marriage in the past year	2.6 (98)	2.3 (31)	2.5 (42)	3.3 (25)

a *Multiple answers were permitted*.

**Table 2:** Depression-, anxiety- and hostility symptoms by survey year (1985, 1988, 1991).

		**Descriptive values**			**Change score**		
**Survey year**	***n***	**Range^a^**	**Mean (SD)**	**Median (IQR)^b^**	**Estimator^c^ (95% CI)**	***p*****-value**	***p*****-value (global)**
**Depression**							
1985	1,437	0–3.00	0.21 (0.43)	0.00 (0, 0.33)			
1988	1,233	0–4.00	0.22 (0.42)	0.00 (0, 0.33)	0.02 (0, 0.03)	0.08	0.99
1991	1,099	0–3.17	0.22 (0.43)	0.00 (0, 0.33)	0.02 (0, 0.04)	0.13	
**Anxiety**							
1985	1,437	0–3.83	0.22 (0.38)	0.00 (0, 0.33)			
1988	1,229	0–3.83	0.21 (0.37)	0.00 (0, 0.33)	0.00 (−0.02, 0.01)	0.529	0.014
1991	1,116	0–3.50	0.22 (0.37)	0.00 (0, 0.33)	0.02 (0.01, 0.04)	0.006	
**Hostility**							
1985	1,437	0–3.80	0.26 (0.39)	0.20 (0, 0.40)			
1988	1,232	0–3.80	0.24 (0.35)	0.20 (0, 0.40)	−0.01 (−0.02, 0.01)	0.480	0.613
1991	1,110	0–3.60	0.24 (0.38)	0.20 (0, 0.40)	0.00 (−0.02, 0.02)	0.956	

a *Minimum-Maximum*,

b *Inter quartile range (25% percentile −75% percentile)*,

c *Baseline and age adjusted*.

**Figure 1:**
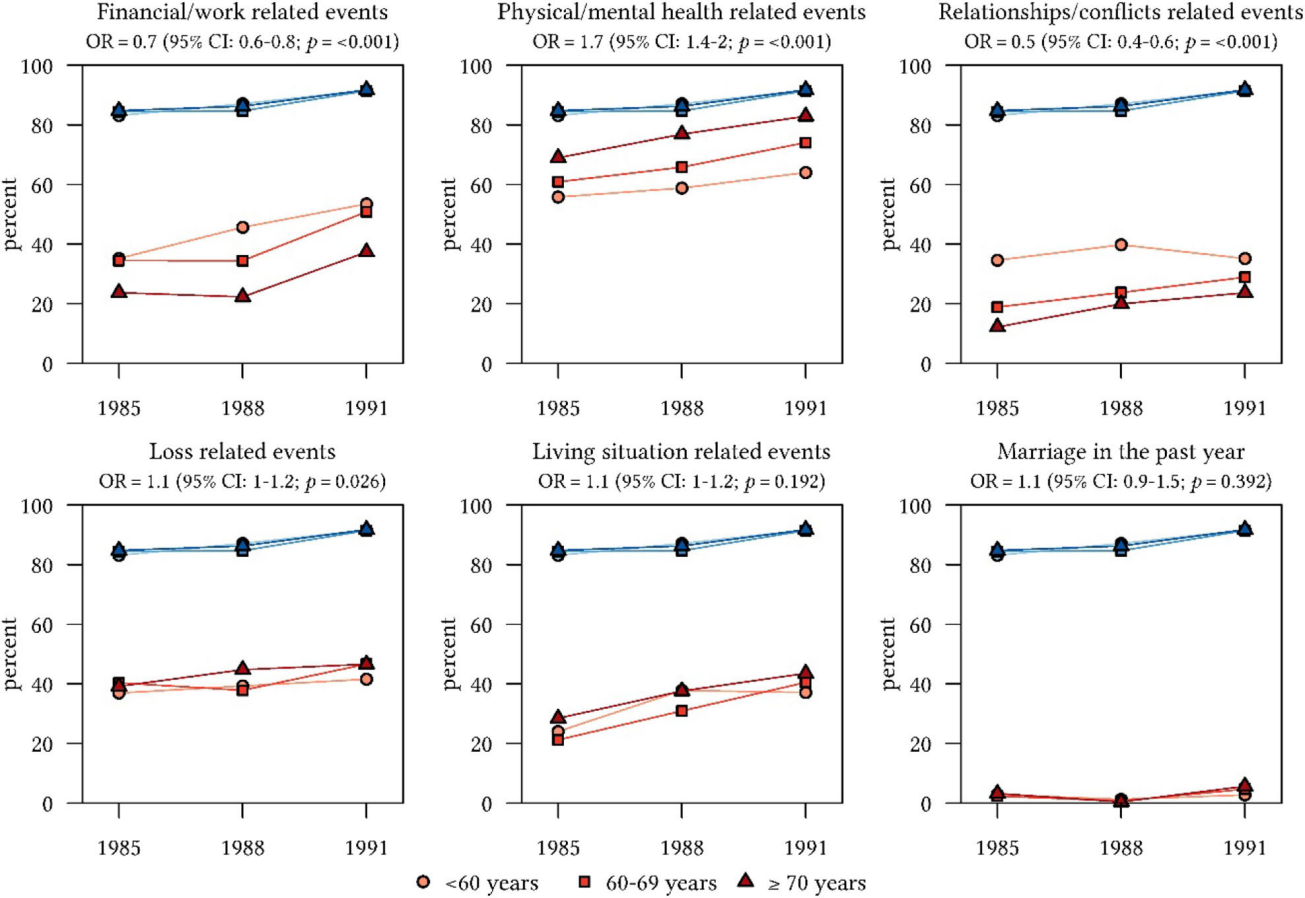
Frequency (%) of stessful life events (SLEs) by age categories at three time points in relation to all reported life events (blue lines).

Figure 1 suggests differences in SLEs between age categories and across survey years. Overall, 86.8% of participants reported at least one SLE across the three survey waves. The probability of SLEs does not change significantly by baseline age, adjusted for the assessment year but increases significantly over the years from 1985 to 1991 (*p*-value for linear trend: <0.001). The most frequently reported SLE was “Deterioration of memory,” with 51.1 % (across all age categories and years). Reports on “Relationships-related events” and on “Financial/work-related events” decreased significantly with increasing age, while the probability of “health- and loss- related events” increased with increasing age. Figure 1 (Graphs 1-6) presents the ORs and corresponding 95%-CIs according to age across the three survey years.

### Depression, Anxiety and Hostility Over 6 Years

We estimated depression-, anxiety- and hostility symptoms over 6 years. Mean values of depression, anxiety and hostility symptoms remained relatively unchanged over this period in these men living in the Boston area.

#### Effects of Stressful Life Events on Depression, Anxiety and Hostility Symptoms

To estimate the impact of SLEs on depression, anxiety and hostility symptoms by number and type of SLEs, baseline-, and age-adjusted symptoms were estimated (Table 3). Quantity of SLEs was significantly associated with changes in depression, anxiety and hostility symptoms. The occurrence of at least one SLE in the past year had significant impact on depression, anxiety, and hostility symptoms.

**Table 3:**
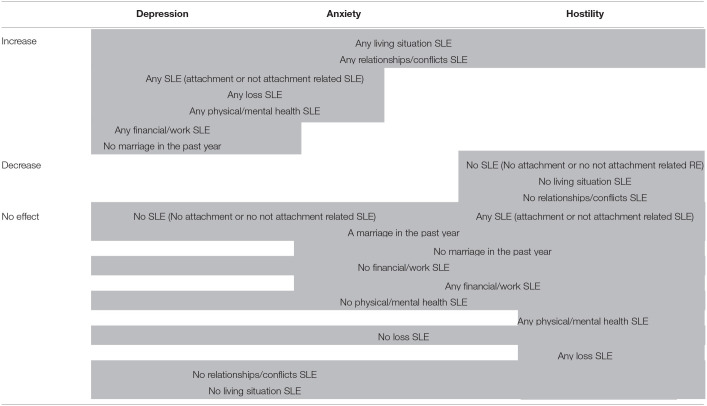
Effects of stressful life events on depression, anxiety and hostility.

**Figure 2:**
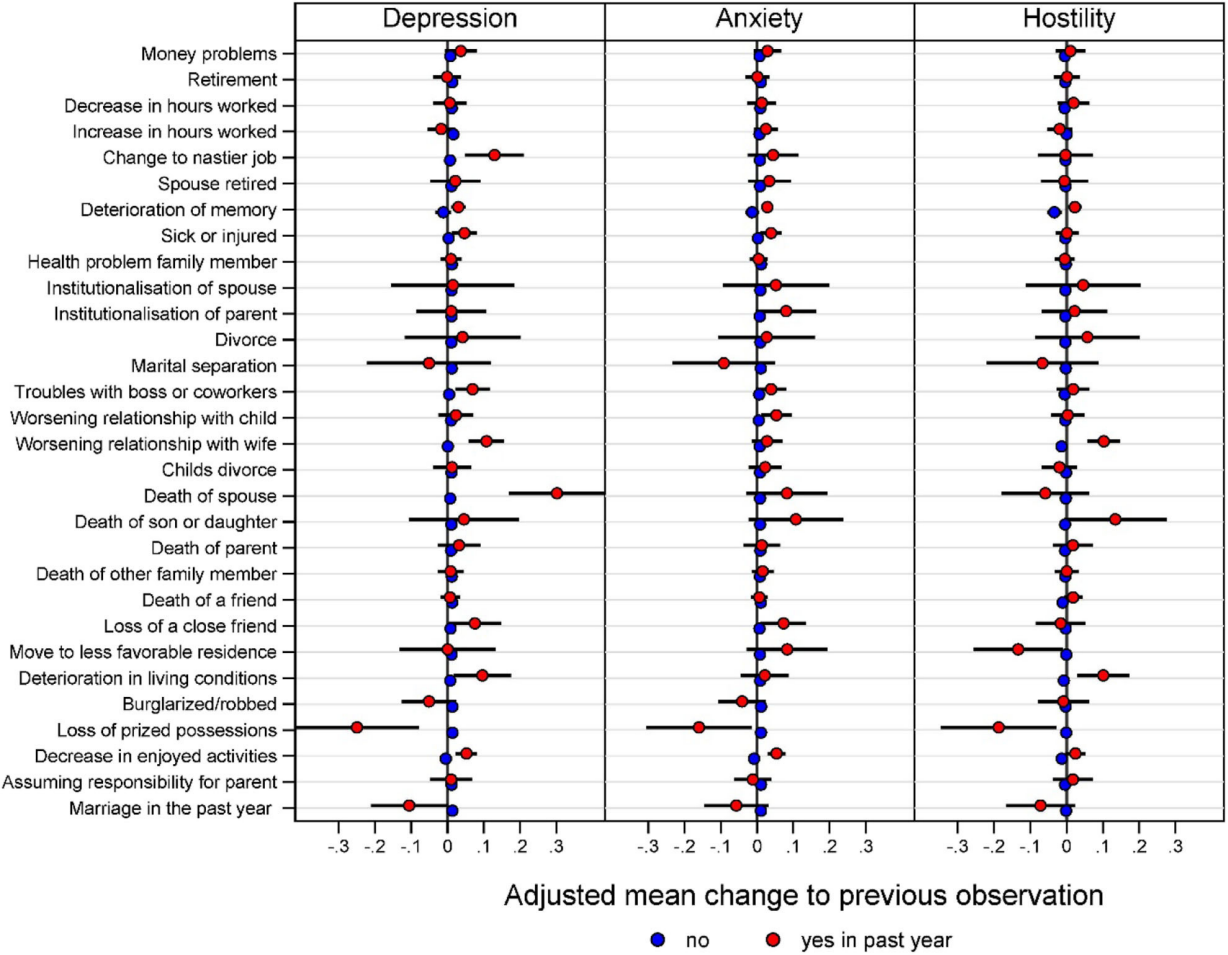
Estimated changes to previous assessment for SLEs and depression-, anxiety- and hostility symptoms in a multilevel mixed-effects linear regression model adjusted for age, baseline and all other SLEs.

Figure 2 displays the effects of individual SLEs on depression-, anxiety- and hostility symptom levels. Negative relationship-events (“worsening relationship with wife, “death of spouse; “loss of a close friend) were significantly associated with increased scores of depression and anxiety symptoms (“worsening relationship with child”; “loss of a close friend”). Furthermore, negative social interactions (“worsening relationship with wife,” “deterioration of living conditions” and “deterioration of memory”) were statistically significant associated with increases in hostility The relationship event marriage in the past year was associated with significantly less symptoms of depression, anxiety and hostility.

#### Effects of SLE Categories on Changes in Depression, Anxiety and Hostility Over 6 Years

Table 4 presents the effects of types of SLEs on changes in depression, anxiety and hostility symptoms comparing the effect of categories of SLEs vs. no SLEs. Financial/work related events, health and loss related events had no effect on depression-, anxiety- or anger symptom changes. On the contrary, negative relationship- and living situation related events had a significant effect on symptom changes.

**Table 4:** Effects of SLE categories on changes of depression-, anxiety- and hostility symptoms.

**SLE**	**Depression**	**Anxiety**	**Hostility**
Any	+0.05^*^	+0.04^*^	+0.05^*^
Negative social interactions	+0.07^*^	+0.03^*^	+0.06^*^
Living situation	+0.07^*^	+0.05^*^	+0.06^*^
Health	*+0,03 n.s*.	+0.03	+*0.02 n.s*.
Marriage	*−0.05 n.s*.^**^	*−0.05 n.s*.	*−0.07 n.s*.
Finances	*+0.01 n.s*.	*+0.02 n.s*.	*+0.03 n.s*.
Loss	*+0.01 n.s*.	*+0.02 n.s*.	*+0.02 n.s*.

*n.s., = not significant*.

* *+= Increases in symptoms*;

** *−= decreases in symptoms*.

*All changes were adjusted for age and baseline scores of depression, anxiety and hostility*.

## Discussion

The aim of this study was to investigate the impact of individual SLEs on depression, anxiety, and hostility in older men and to identify events associated with an impact compared to no events and in comparison to all other events. In sum, we found that quantity of SLEs was associated with increases in depression-, anxiety- and hostility symptoms. Moreover, relationship-related events were associated with significant increases in depression-, anxiety-, and hostility symptoms among men in this study. However, our findings suggest that, depression, anxiety, and hostility symptoms in this population are relatively stable in the absence of relationship-related SLEs.

Our findings confirmed that the amount of stressful life events is associated with depression in line with existing evidence on the relationship between SLEs and depressive symptoms (19, 34). Our study extends previous research on associations of SLEs and depression and anxiety. We find that older men are more affected by negative social interactions than by any other events, suggesting that older individuals find it especially difficult to cope with negative social interactions and with changes in the living situation. Relationships and the living situation allow feelings of belonging to persons and/or to places. Our results are consistent with the continuity theory of aging, which posits that older individuals attempt to preserve and maintain relationships, living situations, and lifestyles as much as possible as they grow older (35).

This study reveals that negative social interactions have an impact on changes in depression-, anxiety-, and hostility symptoms. We found more problems with depression, anxiety and hostility among those with negative social interactions. Our findings are in line with a study on community dwelling individuals aged 65± living in London suggesting that lack of contact with friends and low levels of social support contribute to maintenance of depression among older individuals (36). With regards to supportive personal relations, other work has suggested that supportive personal relations reduce psychopathological symptoms (37). However, in this study the effects of negative social interactions were small. It might be that this cohort is especially resilient and relatively unaffected by SLEs but negative relationships are more important than any other events, even in this relatively resilient cohort of men. Accordingly, there should be a note of caution regarding the certainty with which practical implications for real-world action might be taken from these findings. Yet, it might be, that in other cohorts of less resilient persons, effects of negative social interactions are even stronger. However, our findings strongly suggest that interventions to prevent and reduce depression, anxiety and anger in older people need to be designed with regard to the importance of negative social interactions. It might be necessary to reduce negative social interactions among (older) people, rather than focusing on the needs of practical and task-oriented support. Interventions which address depression, anxiety and hostility in older people might specifically evaluate the relationships of older people.

We acknowledge some limitations of the present study. First, the sample is a male cohort, predominantly white, and results may differ in samples with women and greater ethnic diversity. SLEs in this study were defined according to self-report from a limited list of “present/absent” events. The validity of such scales relies on the extent to which events are reported. This in turn depends on participants being able to recall, and willing to report them. However, cognitive impairment—to an extent that would influence the ability to recall important previous or recent events—is unlikely to be an important factor in this sample because the recall time is short. A further limitation might be that men with better health might have remained in the cohort as in most longitudinal studies. Despite these limitations, our study has several methodological strengths. Notably, the study used a large, prospective, sample of men living in the Greater Boston, MA, United States area. It allows for some establishment of temporal precedence, lending confidence to the notion that social relationship related-related events have an impact on depression-, anxiety- and hostility symptoms in aging men.

Social interactions and belonging to persons and places cannot easily be measured but it seems to be a promising field of research. Studies on social interactions might be even more necessary given that depression might be associated with the risk of incident cognitive decline among older participants (38). Our results showed that negative relationship events were associated with significant changes in depression, anxiety- and hostility symptoms. Our findings might be particular relevant during the time of the COVID-19 pandemic when elder individuals might suffer from negative life events and social isolation due to the pandemic. These negative life events and social isolation might contribute to depression, anxiety and hostility among the affected persons. Developing interventions for those with negative life events and social isolation might be crucial. Future studies are necessary to investigate the mechanisms why relationship events are more important in the aging population than any other stressful life event.

## Data Availability Statement

The raw data supporting the conclusions of this article will be made available by the authors, without undue reservation.

## Ethics Statement

The studies involving human participants were reviewed and approved by VA Institutional Review Board. The patients/participants provided their written informed consent to participate in this study.

## Author Contributions

JL and AS: study design. LOL and AS: data collection. JL and SS: analysis. JL, MGW, LOL, AS, and MM: interpretation. JL: draft of the manuscript. LOL, MGW, MM, and AS: critical revision of the manuscript. All authors: approval of the final version for publication.

## Conflict of Interest

The authors declare that the research was conducted in the absence of any commercial or financial relationships that could be construed as a potential conflict of interest.
