# Supplementary material for: Threats to Belonging—Stressful Life Events and Mental Health Symptoms in Aging Men—A Longitudinal Cohort Study
Source: Front Psychiatry. 2020 Dec 2;11:575979. doi: 10.3389/fpsyt.2020.575979 (PMC7793980; doi:10.3389/fpsyt.2020.575979)
Supplement: Supplementary file 1 [file Data_Sheet_1.docx]

**Supplemental material**

**Table 1. Descriptive characteristics of the population by survey year**

|  | **1985** |  | **1988** |  | **1991** |  |
| --- | --- | --- | --- | --- | --- | --- |
|  | **n** | **%** | **n** | **%** | **n** | % |
| Retired | 461 | 32.1 | 497 | 39.3 | 591 | 40.2 |
| Retired (working part- time) | 155 | 10.8 | 186 | 14.7 | 179 | 15.5 |
| Retired (working full-time) | 70 | 4.9 | 83 | 6.6 | 62 | 5.4 |
| Work (part-time) | 27 | 1.9 | 23 | 1.8 | 13 | 1.1 |
| Work (full-time) | 692 | 48.2 | 449 | 35.5 | 270 | 23.4 |
| Unemployed | 15 | 1.0 | 5 | 0.4 | 13 | 1.1 |
| Missing | 17 | 1.2 | 22 | 1,7 | 24 | 2.1 |

**Table 2. Stressful life event by age group (financial/work -, health -, relationship -, loss -, living situation events and marriage) in the past year**

| **Type of SLE^a^** | | **Age categories at baseline** | | | |
| --- | --- | --- | --- | --- | --- |
|  |  | **Total** | **<59** | **60-69** | **≥70** |
|  |  | %  (n) | %  (n) | %  (n) | %  (n) |
| **Any SLE** | | 86.8  (3328) | 86.0  (1157) | 86.8  (1467) | 88.2  (704) |
| **Financial / work events** | | 38.3  (1462) | 41.9  (563) | 39.7  (670) | 29.0  (229) |
|  | Money problems | 9.6  (366) | 11.0  (147) | 8.9  (149) | 9.0  (70) |
|  | Retirement | 14.5  (548) | 9.3  (125) | 19.4  (324) | 12.9  (99) |
|  | Decrease in hours worked | 9.4  (356) | 9.7  (130) | 10.0  (166) | 7.8  (60) |
|  | Increase in hours worked | 14.7  (553) | 22.5  (301) | 11.7  (194) | 7.5  (58) |
|  | Change to worse job | 3.4  (129) | 5.5  (73) | 2.9  (48) | 1.0  (8) |
|  | Spouse retired | 4.2  (160) | 2.7  (36) | 5.9  (99) | 3.2  (25) |
| **Health events** | | 66.01 (2530) | 58.3  (784) | 66.8  (1128) | 77.5  (618) |
|  | Deterioration of memory | 51.1  (1942) | 40.4  (542) | 52.6  (880) | 65.9  (520) |
|  | Sick or injured | 17.1  (649) | 14.1  (189) | 17.9  (300) | 20.5  (160) |
|  | Health problem family member | 26.5  (1006) | 25.6  (343) | 25.6  (429) | 30.0  (234) |
|  | Institutionalisation of spouse | 1.1  (41) | 0.5  (7) | 1.0  (16) | 2.3  (18) |
|  | Institutionalisation of parent | 2.3  (88) | 3.7  (49) | 2.0  (33) | 0.8  (6) |
| **Relationship events** | | **27.3**  **(1042)** | **36.3**  **(487)** | **23.8**  **(400)** | **19.6**  **(155)** |
|  | Divorce | 1.1  (42) | 1.6  (22) | 0.9  (15) | 0.6  (5) |
|  | Marital separation | 1.4  (53) | 2.1  (28) | 0.9  (15) | 1.3  (10) |
|  | Troubles with boss or coworkers | 10.3  (390) | 20.1  (270) | 6.7  (112) | 1.0  (8) |
|  | Worsening relationship with child | 8.7  (329) | 11.8  (158) | 7.6  (128) | 5.5  (43) |
|  | Worsening relationship with wife | 8.9  (335) | 10.2  (137) | 7.4  (124) | 9.5  (74) |
|  | Childs divorce | 7.3  (275) | 6.0  (81) | 8.6  (143) | 6.6  (51) |
| **Loss events** | | **41.0**  **(1568)** | **38.5**  **(518)** | **41.5**  **(701)** | **44.2**  **(349)** |
|  | Death of spouse | 1.7  (64) | 0.5  (7) | 1.1  (18) | 5.0  (39) |
|  | Death of son or daughter | 0.7  (28) | 0.6  (8) | 0.8  (13) | 0.9  (7) |
|  | Death of parent | 5.7  (215) | 6.7  (90) | 5.5  (92) | 4.3  (33) |
|  | Death of other family member | 16.6  (627) | 14.0  (187) | 18.5  (309) | 16.9  (131) |
|  | Death of a friend | 27.1  (1022) | 22.7  (305) | 28.7  (477) | 31.4  (240) |
|  | Loss of a close friend | 3.7  (140) | 4.9  (66) | 2.6  (43) | 4.0  (31) |
| **Living situation related events** | | **32.2**  **(1232)** | **30.7**  **(413)** | **30.8**  **(518)** | **37.9**  **(301)** |
|  | Move to worse residence | 1.8  (67) | 2.5  (33) | 1.6  (26) | 1.0  (8) |
|  | Deterioration in living conditions | 3.8  (146) | 4.1  (55) | 4.1  (69) | 2.8  (22) |
|  | Burglarized/robbed | 3.6  (138) | 4.1  (55) | 3.8  (63) | 2.6  (20) |
|  | Loss of prized possessions | 1.1  (40) | 1.7  (23) | 0.6  (10) | 0.9  (7) |
|  | Decrease in enjoyed activities | 24.1  (917) | 18.4  (246) | 23.8  (399) | 34.6  (272) |
|  | Assuming responsibility for parent | 6.6  (250) | 10.1  (135) | 5.7  (96) | 2.4  (19) |
| **Marriage in the past year** | | **2.6**  **(98)** | **2.3**  **(31)** | **2.5**  **(42)** | **3.3**  **(25)** |

^1^ multiple answers were permitted

**Table 3. Type of stressful life event over all three measuring times by age group (defined by baseline age) clustered into six groups (financial/work related events, physical/mental health related events, relationships/conflicts related events, loss related events, living situation related events and marriage in the past year).**

| **Type of SLE^a^** | | **Age groups defined by baseline age** | | | |
| --- | --- | --- | --- | --- | --- |
|  |  | **Total** | **<59** | **60-69** | **≥70** |
|  |  | **%**  **(n)** | **%**  **(n)** | **%**  **(n)** | **%**  **(n)** |
| **Any SLE** | | **86.8**  **(3328)** | **86.0**  **(1157)** | **86.8**  **(1467)** | **88.2**  **(704)** |
| **Financial / work related events** | | **38.3**  **(1462)** | **41.9**  **(563)** | **39.7**  **(670)** | **29.0**  **(229)** |
|  | Money problems | 9.6  (366) | 11.0  (147) | 8.9  (149) | 9.0  (70) |
|  | Retirement | 14.5  (548) | 9.3  (125) | 19.4(324) | 12.9  (99) |
|  | Decrease in hours worked | 9.4  (356) | 9.7  (130) | 10.0  (166) | 7.8  (60) |
|  | Increase in hours worked | 14.7  (553) | 22.5  (301) | 11.7  (194) | 7.5  (58) |
|  | Change to worse job | 3.4  (129) | 5.5  (73) | 2.9  (48) | 1.0  (8) |
|  | Spouse retired | 4.2  (160) | 2.7  (36) | 5.9  (99) | 3.2  (25) |
| **Physical / mental health related events** | | **66.01 (2530)** | **58.3**  **(784)** | **66.8**  **(1128)** | **77.5**  **(618)** |
|  | Deterioration of memory | 51.1  (1942) | 40.4  (542) | 52.6  (880) | 65.9  (520) |
|  | Sick or injured | 17.1  (649) | 14.1  (189) | 17.9  (300) | 20.5  (160) |
|  | Health problem family member | 26.5  (1006) | 25.6  (343) | 25.6  (429) | 30.0  (234) |
|  | Institutionalisation of spouse | 1.1  (41) | 0.5  (7) | 1.0  (16) | 2.3  (18) |
|  | Institutionalisation of parent | 2.3  (88) | 3.7  (49) | 2.0  (33) | 0.8  (6) |
| **Relationship related events** | | **27.3**  **(1042)** | **36.3**  **(487)** | **23.8**  **(400)** | **19.6**  **(155)** |
|  | Divorce | 1.1  (42) | 1.6  (22) | 0.9  (15) | 0.6  (5) |
|  | Marital separation | 1.4  (53) | 2.1  (28) | 0.9  (15) | 1.3  (10) |
|  | Troubles with boss or coworkers | 10.3  (390) | 20.1  (270) | 6.7  (112) | 1.0  (8) |
|  | Worsening relationship with child | 8.7  (329) | 11.8  (158) | 7.6  (128) | 5.5  (43) |
|  | Worsening relationship with wife | 8.9  (335) | 10.2  (137) | 7.4  (124) | 9.5  (74) |
|  | Childs divorce | 7.3  (275) | 6.0  (81) | 8.6  (143) | 6.6  (51) |
| **Loss related events** | | **41.0**  **(1568)** | **38.5**  **(518)** | **41.5**  **(701)** | **44.2**  **(349)** |
|  | Death of spouse | 1.7  (64) | 0.5  (7) | 1.1  (18) | 5.0  (39) |
|  | Death of son or daughter | 0.7  (28) | 0.6  (8) | 0.8  (13) | 0.9  (7) |
|  | Death of parent | 5.7  (215) | 6.7  (90) | 5.5  (92) | 4.3  (33) |
|  | Death of other family member | 16.6  (627) | 14.0  (187) | 18.5  (309) | 16.9  (131) |
|  | Death of a friend | 27.1  (1022) | 22.7  (305) | 28.7  (477) | 31.4  (240) |
|  | Loss of a close friend | 3.7  (140) | 4.9  (66) | 2.6  (43) | 4.0  (31) |
| **Living situation related events** | | **32.2**  **(1232)** | **30.7 (413)** | **30.8**  **(518)** | **37.9**  **(301)** |
|  | Move to worse residence | 1.8  (67) | 2.5  (33) | 1.6  (26) | 1.0  (8) |
|  | Deterioration in living conditions | 3.8  (146) | 4.1  (55) | 4.1  (69) | 2.8  (22) |
|  | Burglarized/robbed | 3.6  (138) | 4.1  (55) | 3.8  (63) | 2.6  (20) |
|  | Loss of prized possessions | 1.1  (40) | 1.7  (23) | 0.6  (10) | 0.9  (7) |
|  | Decrease in enjoyed activities | 24.1  (917) | 18.4  (246) | 23.8  (399) | 34.6  (272) |
|  | Assuming responsibility for parent | 6.6  (250) | 10.1  (135) | 5.7  (96) | 2.4  (19) |
| **Marriage in the past year** | | **2.6**  **(98)** | **2.3**  **(31)** | **2.5**  **(42)** | **3.3**  **(25)** |

^1^ Multiple answers were permitted

**Table 4. Depression-, anxiety- and hostility symptoms by survey year (1985, 1988, 1991)**

|  | | **Descriptive values** | | | **Change score** | | |
| --- | --- | --- | --- | --- | --- | --- | --- |
| **Survey year** | **n** | **Range^a^** | **Mean**  **(SD)** | **Median**  **(IQR)^b^** | **Estimator^c^**  (**95%CI**) | **p-value** | **p-value**  **(_global)_** |
| **Depression symptoms** | | | | | | | |
| 1985 | 1437 | 0-3.00 | 0.21  (0.43) | 0.00  (0-0.33) |  | | |
| 1988 | 1233 | 0-4.00 | 0.22  (0.42) | 0.00  (0-0.33) | 0.02  (0-0.03) | 0.08 | 0.99 |
| 1991 | 1099 | 0-3.17 | 0.22  (0.43) | 0.00  (0-0.33) | 0.02  (0-0.04) | 0.13 |  |
| **Anxiety symptoms** | | | | | | | |
| 1985 | 1437 | 0-3.83 | 0.22  (0.38) | 0.00  (0-0.33) |  | | |
| 1988 | 1229 | 0-3.83 | 0.21  (0.37) | 0.00  (0-0.33) | 0.00  (-0.02-0.01) | 0.529 | 0.014 |
| 1991 | 1116 | 0-3.50 | 0.22  (0.37) | 0.00  (0-0.33) | 0.02  (0.01-0.04) | 0.006 |  |
| **Hostility symptoms** | | | | | | | |
| 1985 | 1437 | 0-3.80 | 0.26  (0.39) | 0.20  (0-0.40) |  | | |
| 1988 | 1232 | 0-3.80 | 0.24  (0.35) | 0.20  (0-0.40) | -0.01  (-0.02-0.01) | 0.480 | 0.613 |
| 1991 | 1110 | 0-3.60 | 0.24  (0.38) | 0.20  (0-0.40) | 0.00  (-0.02-0.02) | 0.956 |  |

a Minimum-Maximum, b Inter quartile range (25% percentile -75% percentile), c Baseline and age adjusted.

**Table 5. Estimated changes in depression, anxiety and hostility for groups of SLEs, multilevel mixed-effects linear regression adjusted for age, baseline and all other SLEs**

| **SLE** | | **Depression** | | **Anxiety** | | **Hostility** | |
| --- | --- | --- | --- | --- | --- | --- | --- |
|  |  | **Estimated change**  **(95% CI)** | ***p-value*** | **Estimated change**  **(95% CI)** | ***p-value*** | **Estimated change**  **(95% CI)** | ***p-value*** |
| **Any SLE** | | | | | | | |
| Any | no | -0.03 (-0.07,-0.01) | .108 | -0.03 (-0.06,-0.01) | .108 | -0.05 (-0.08,-0.01) | .012 |
|  | yes | 0.02 (0.01,-0.04) | .002 | 0.01 (0.00,-0.02) | .043 | 0.00 (-0.01,-0.02) | .692 |
| **Categories of SLEs vs. no-SLEs** | | | | | | | |
| Financial / work | no | 0.01 (-0.01, 0.03) | .310 | 0.00 (-0.01-0.02) | .823 | -0.01 (-0.03,-0.00) | .142 |
|  | yes | 0.02 (0.00,-0.05) | .033 | 0.02 (-0.00,-0.04) | .053 | 0.01 (-0.01,-0.03) | .205 |
| Health | no | -0.01 (-0.03,-0.02) | .683 | -0.01 (-0.03,-0.01) | .371 | -0.02 (-0.04,-0.00) | .120 |
|  | yes | 0.02 (0.01,-0.04) | .004 | 0.02 (0.00,-0.03) | .019 | 0.01 (-0.01,-0.02) | .486 |
| Relations | no | 0.00 (-0.02,-0.01) | .594 | 0.00 (-0.01,-0.01) | .944 | -0.02 (-0.03,-0.00) | .016 |
|  | yes | 0.06 (0.04,-0.09) | <.001 | 0.03 (0.01,-0.05) | .011 | 0.04 (0.01,-0.06) | .002 |
| Loss | no | 0.01 (-0.01,-0.03) | .297 | 0.00 (-0.01,-0.02) | .806 | -0.01 (-0.03,-0.01) | .302 |
|  | yes | 0.02 (0.00,-0.04) | .029 | 0.02 (0.00,-0.04) | .049 | 0.01 (-0.01,-0.03) | .469 |
| Living situation | no | -0.01 (-0.03,-0.01) | .286 | -0.01 (-0.03,-0.00) | .121 | -0.03 (-0.04,-0.01) | .002 |
|  | yes | 0.06 (0.03,-0.08) | <.001 | 0.04 (0.02,-0.06) | <.001 | 0.04 (0.02,-0.06) | .002 |
| Marriage | no | 0.02 (0.00,-0.03) | .017 | 0.01 (-0.00,-0.02) | .092 | 0.00 (-0.01,-0.01) | .965 |
|  | yes | -0.04 (-0.12,-0.05) | .387 | -0.04 (-0.11,-0.03) | .256 | -0.07 (-0.15,-0.01) | .094 |
| **Relationship related events vs. all other** | | | | | | | |
| Relationship related events | no | -0.01 (-0.03-0.01) | .259 | -0.01 (-0.03-0.01) | .418 | -0.02 (-0.04- -0.00) | .018 |
|  | yes | 0.03 (0.02-0.05) | <.001 | 0.02 (0.00-0.03) | .014 | 0.01 (-0.00-0.03) | .150 |
| Other than  relationship related events | no | -0.01 (-0.04-0.02) | .371 | -0.02 (-0.04-0.01) | .233 | -0.04 (-0.07- -0.01) | .005 |
|  | yes | 0.02 (0.01-0.04) | .003 | 0.01 (0.00-0.03) | .033 | 0.01 (-0.01-0.02) | .344 |

**Table 6: Effects of SLE categories on changes of depression-, anxiety- and hostility symptoms**

|  | **Depression** | **Anxiety** | **Hostility** |
| --- | --- | --- | --- |
| Any SLE | +0.05* | +0.04* | +0.05* |
| Any relationship SLE | +0.07* | +0.03* | +0.06* |
| Living situation SLE | +0.07* | +0.05* | +0.06* |
| Health SLE | *+0,03 n.s.* | +0.03 | *+0.02 n.s.* |
| Marriage | -0.05 n.s**. | -0.05 n.s. | *-0.07 n.s.* |
| Finances SLE | *+0,01 n.s.* | *+0.02 n.s.* | *+0.03 n.s.* |
| Loss SLE | *+0,01 n.s.* | *+0.02 n.s.* | *+0.02 n.s.* |

*+=increases in symptoms; ** -=decreases in symptoms.

Note. All changes were adjusted for age and baseline scores of depression, anxiety and hostility.
